# c-Jun-mediated miR-19b expression induces endothelial barrier dysfunction in an in vitro model of hemorrhagic shock

**DOI:** 10.1186/s10020-022-00550-0

**Published:** 2022-10-12

**Authors:** Feng Wu, Jian-Ying Wang, Brooke Dorman, Ahmad Zeineddin, Rosemary Ann Kozar

**Affiliations:** 1 Shock Trauma Center, University of Maryland School of Medicine, Baltimore, MD USA; 2 Department of Surgery, University of Maryland School of Medicine, Baltimore, MD USA

**Keywords:** Lung microvascular endothelial cells, Hypoxia/reoxygenation, miR-19b, c-Jun, Endothelial barrier

## Abstract

**Background:**

Our previous data demonstrated that miR-19b expression was increased in human lung microvascular endothelial cells in-vitro*-, *in-vivo and in patients with hemorrhagic shock, leading to a decrease in syndecan-1 mRNA and protein and resulting in loss of endothelial barrier function. However, the mechanism underlying increased miR-19b expression remains unclear. The objective of the current study was to determine if c-Jun mediates the early responsive microRNA, miR-19b, to cause endothelial barrier dysfunction.

**Method:**

Human lung microvascular endothelial cells (HLMEC) or HEK293T cells were transfected with c-Jun overexpressing vector, c-Jun siRNA, miR-19b promoter vector, miR-19b mutated promoter vector, miR-19b oligo inhibitor, then subjected to hypoxia/reoxygenation as in-vitro model of hemorrhagic shock. Levels of protein, miRNA, and luciferase activity were measured. Transwell permeability of endothelial monolayers were also determined. Plasma levels of c-Jun were measured in injured patients with hemorrhagic shock.

**Result:**

Hypoxia/reoxygenation induced primary (pri-)miR-19b, mature miR-19b, and c-Jun expression over time in a comparable timeframe. c-Jun silencing by transfection with its specific siRNA reduced both pri-miR-19b and mature miR-19b levels. Conversely, c-Jun overexpression enhanced H/R-induced pri-miR-19b. Studies using a luciferase reporter assay revealed that in cells transfected with vectors containing the wild-type miR-19b promoter and luciferase reporter, c-Jun overexpression or hypoxia/ reoxygenation significantly increased luciferase activity. c-Jun knockdown reduced the luciferase activity in these cells, suggesting that the miR-19b promoter is directly activated by c-Jun. Further, chromatin immunoprecipitation assay confirmed that c-Jun directly bound to the promoter DNA of miR-19b and hypoxia/reoxygenation significantly increased this interaction. Additionally, c-Jun silencing prevented cell surface syndecan-1 loss and endothelial barrier dysfunction in HLMECs after hypoxia/reoxygenation. Lastly, c-Jun was significantly elevated in patients with hemorrhagic shock compared to healthy controls.

**Conclusion:**

Transcription factor c-Jun is inducible by hypoxia/reoxygenation, binds to and activates the miR-19b promoter. Using an in-vitro model of hemorrhagic shock, our findings identified a novel cellular mechanism whereby hypoxia/ reoxygenation increases miR-19b transcription by inducing c-Jun and leads to syndecan-1 decrease and endothelial cell barrier dysfunction. This finding supports that miR-19b could be a potential therapeutic target for hemorrhage shock.

**Supplementary Information:**

The online version contains supplementary material available at 10.1186/s10020-022-00550-0.

## Introduction

miRNAs have been linked to endothelial cell dysfunction (Zheng et al. 2021; Chang et al. 2021). We have been interested in the molecular mechanisms regulating endothelial cell dysfunction after hemorrhagic shock (Wu et al. 2021; Chipman et al. 2021) and identified miR-19b as a key molecule (Wu et al. 2020). miR-19b belongs to the miR-17-92 family of miRNA clusters. This miRNA has been shown to play a role in cancer and to be associated with both acute inflammatory conditions such as sepsis as well as chronic inflammatory conditions such as diabetes, rheumatoid arthritis and atherosclerosis (Hu et al. 2018; Li et al. 2018; lv et al. 2014; Niu et al. 2018). Li et al. demonstrated elevated systemic levels of miR-19b in patients with unstable angina and documented its endothelial cell origin (Li et al. 2014; Li et al. 2019).

Endothelial cell dysfunction can be caused by oxidative stress from hypoxia and is a key mechanism of cellular damage. We have previously demonstrated miR-19b inhibited syndecan-1, leading to lung vascular leakage after hemorrhage shock (Wu et al. 2021; Chipman et al. 2021). Additionally, miR-19b inhibition attenuated inflammation and vascular leakage in shock lungs (Wu et al. 2020; Chipman et al. 2021). These results support that miR-19b is a pro-inflammatory miRNA that leads to endothelial barrier dysfunction. However, the exact mechanism by which miR-19b expression is regulated remains unknown.

During hypoxia/oxygenation in-vitro or hemorrhagic shock in-vivo, hypoxia triggers changes in metabolism, the intracellular redox state, and the expression of acute phase proteins (de Jager et al. 2021; Sims and Baur 2017). Reperfusion of ischemic tissues is then associated with an induction in reactive oxygen species (ROS) that cause direct cell damage by oxidation of cellular components (Sims et al. 2017), and indirectly through the activation of early responsive genes, such as c-Jun N-terminal kinases (JNK) (Yang et al. 2013; Relja et al. 2009). Following activation, JNK translocate into the nucleus where they physically associate with and activate their targets, one of which is c-Jun. c-Jun is a member of the Jun family of proteins that are primary components of the activating protein (AP-1) transcription factor (Paxian et al. 2002; Relja et al. 2009). AP-1 regulates a number of miRNAs by directly binding to their target promoters at specific DNA elements (Zhang et al. 2018; Zhong et al. 2019). Although c-Jun, JunB, and JunD have similar DNA binding affinity, their expression patterns vary, with c-Jun being an immediate early response gene that has been implicated in organ damage and inflammation following hemorrhagic shock (Dosch and Kaina 1996; Relja et al. 2009). In the current study, we sought to investigate if c-Jun induces miR-19b early responsive expression to mediate endothelial barrier dysfunction.

## Materials and methods

### Primary cell culture

Human lung microvascular endothelial cells (HLMEC; Sigma) were grown to confluence in endothelial basic medium-2 (EBM-2; Lonza) supplemented with 5% FBS, human recombinant epidermal growth factor, human recombinant insulin-like growth factor-1, human basic fibroblast growth factor, vascular endothelial growth factor, hydrocortisone, ascorbic acid, heparin, gentamicin, and amphotericin B. Endothelial cells (*passages 5–10*) were used for following experiments.

### Hypoxia/reoxygenation (H/R)

H/R was used as an in-vitro model of hemorrhagic shock and was conducted as we described previously (Wu et al. 2021). For normoxia, cells were cultured in EBM-2. For H/R, cells were cultured in EBM2 and exposed to 94% N_2_, 1% oxygen, and 5% CO_2_ for 18 h followed by normoxia for indicated periods of time in the figure legends.

### Western blotting

Endothelial cells were lysed in NuPAGE LDS samples buffer (Thermo Scientific) for Western blot analysis using antibodies including anti-c-Jun (#9165, Cell Signaling Technology). Blots were also probed with anti-GAPDH antibody (PA1-987, Thermo Scientific) for the reference of sample loading.

### RNA extraction and quantitative real-time PCR

Total RNA was extracted from cells using Trizol reagent and was reversely transcribed using Qiagen miScript RT kit. Real-time PCR was performed using Qiagen miScript SYBR Green PCR kit and miR-19b-3p miScript Primer (Qiagen). Pri-miR-19b (hsa-mir-92a-1 primer assay) and mature miR-19b primer was obtained from Taqman. RNU6 miScript Primer (Qiagen) was used as an endogenous control. Relative RNA amount was calculated using the 2^−ΔΔCt^ method.

### siRNA and miRNA oligo inhibitor transfection

HLMECs were seeded in 6-well plates and grown for 24 h in antibiotic-free EBM-2 containing 5% FBS and supplements. Cells were then transiently transfected by incubation with 100 nM miR-19b oligo inhibitor, c-Jun siRNA, or scrambled RNA (scRNA) and 2.5 ul/ml Lipofectamine 2000 (Thermo Fisher Scientific) in antibiotic-free Opti-MEM for 24 h. The medium was then changed to the growth medium, and the cells were cultured for another 48 h prior to assays. Silencing of the respective proteins was validated by quantitative real-time PCR or Western blot analysis.

### Overexpression of c-Jun

HEK293T cells were transfected with pMIEG3-c-Jun overexpression vector or empty vector (pMIEG3) obtained from Addgen (Watertown, MA) using Lipofectamine 2000 following the manufacturer’s recommended procedures. After 3 day-transfection, the expression of c-Jun-GFP was examined under inverted fluorescent microscopy and images obtained. The cells were further exposed to hypoxia for 18 h or exposed to normoxia only for pri-miR-19b induction.

### Luciferase reporter assay

The full human miR-19b promoter (miR-17-92 gene cluster promoter, positions 5786 to 8494 in accession# NG_032702) was inserted into lentivirus pEZX-LvPG04 dual-reporter vector (GeneCopoeia, MD). This vector uses Gluc (Gaussia Luciferase) as the promoter reporter and SEAP (secreted Alkaline Phosphatase) as the internal control for signal normalization. Mutated miR-19b promoter vector was produced by GeneCopoeia by deleting the two c-Jun binding sites at positions 6237–6243 (TGACTCT) and 7495–7501 (TGTGTCA) in accession# NG_032702 as predicted by PROMO (http://alggen.lsi.upc.es/cgi-bin/promo_v3/promo/promoinit.cgi?dirDB=TF8.3) using 3% dissimilarity in comparison to the c-Jun consensus sequence (TGAC/GTCA). HEK293T cells were transfected with the vectors containing wild-type miR-19b promoter, mutated miR-19b promoter, or empty vectors in 96-well plates. Some HEK293T cells were also co-transfected with pMIEG3-c-Jun overexpression vector (Addgen, MA) to overexpress c-Jun or empty vector (pMIEG3) for negative control. Some HEK293T cells were exposed to hypoxia for 18 h then normoxia for 3 h to induce c-Jun overexpression. The culture medium of the transfected cells was harvested for assays of promoter activity using Secrete-Pair™ Gaussia Luciferase Dual Luminescence Assay Kit (GeneCopoeia). In the assay, the activities of Gluc and SEAP were detected and Gluc activity was normalized to SEAP activity.

### Syndecan-1 immunofluorescence staining

HLMECs were grown directly on 8-well chambers (Corning). Cells were then transiently transfected by incubation with 100 nM c-Jun siRNA or scrambled RNA (scRNA) and 2.5 μl/ml Lipofectamine 2000 in antibiotic-free Opti-MEM for 24 h. The medium was then changed to the growth medium, and the cells were cultured for another 48 h prior to assays. Silencing of the respective proteins was validated by Western blot analysis. After being exposed to hypoxia (94% N_2_, 1% oxygen, and 5% CO_2_) for 18 h, cells were fixed in 4% paraformaldehyde for 15 min and blocked with 2% bovine serum albumin (BSA) in PBS for 1 h at room temperature. Cells were then incubated with anti-syndecan1 antibody (1:100; Cell Signaling) in 1% BSA at 4 °C overnight and followed by incubating with Alexa fluor 488 conjugated anti-mouse IgG (1:200; Invitrogen) in 1% BSA for 2 h at room temperature. The fluorescence intensity was quantified using Quantity One and reported as relative fluorescence units.

### Endothelial barrier integrity

HLMECs were seeded on culture inserts (3 μm pore size, Costar) in 24-well companion plates and grown to confluence in EBM-2 containing 5% FBS and supplements. In some experiments, monolayers were transfected with 100 nM miR-19b oligo inhibitor, c-Jun siRNA, or scRNA before seeding the inserts. Subsequently, FITC-labeled dextran (40 kDa, Sigma) was added to the upper chamber at a concentration of 100 μg/ml, and phosphate buffered saline (PBS) to the lower chamber (to prevent the formation of an oncotic pressure gradient) for 1 h. Medium was collected from the lower chamber, and the fluorescence was measured using a fluorimeter (485 nm excitation, 530 nm emission). The fold change in FITC-dextran fluorescence intensity over controls was used as a measure of monolayer permeability.

### Chromatin immunoprecipitation assay

After exposure to hypoxia for 18 h then normoxia for 3 h, cells were fixed with 1% formaldehyde to crosslink chromatin. Chromatin immunoprecipitation (ChIP) analysis was performed using Piece Magnetic ChIP kit (cat# 26157) and anti-c-Jun antibody (cs#39165, Cell Signaling Technology). The bound-DNA was isolated and purified for quantitative PCR. As described above, the miR-19b promoter contains two c-Jun binding sites at positions 6237–6243 (TGACTCT) and 7495–7501 (TGTGTCA). The primers to detect DNA around position 6237–6243 were 5′-CCTTGTGCGACATGTGCTG -3′ and 5′-GATGGCATGCCGTTAATTTT -3′ (174 bp) and around position 7495–7501 were 5′-GCCACGTGGATGTGAAGATT -3′ and 5′- AAGTGGTGGCTCTTCCAATG -3′ (165 bp). Isotype IgG was used as a negative control. DNA isolated from the whole cell lysates served as input DNA control.

### Human study

Available plasma samples from a recently completed prospective observational clinical study in trauma patients (Zeineddin et al. 2022) were used to measure c-Jun. The present study was approved by the Institutional Review Board of the University of Maryland Baltimore. Informed consent was obtained from all patients and included the consent to investigate biologic markers of endothelial dysfunction in the present study. Samples were de-identified and stored prior to bulk analysis. All experimental procedures were conducted in compliance with the University of Maryland Baltimore and the National Institutes of Health guidelines. This study included severely injured patients in hemorrhage shock, defined as a systolic blood pressure < 90 mm Hg and requiring blood component therapy upon arrival. Plasma was collected at admission in the trauma bay and stored in – 80 °C until time of experiment. For healthy donor controls, aliquots were obtained from 13 random donor units of fresh frozen plasmas obtained from Tennessee Blood Services (Memphis, TN). Plasma c-Jun was measured by ELISA (catalog# NBP2-75279, Novus Biologicals).

### Statistical analysis

Data are expressed as mean ± SE. Values from different groups were analyzed by T test or one-way analysis of variance (ANOVA) with Bonferroni multiple comparison tests with significance set at level at p < 0.05.

## Results

### H/R increased miR-19b and c-Jun expression

**Fig. 1:**
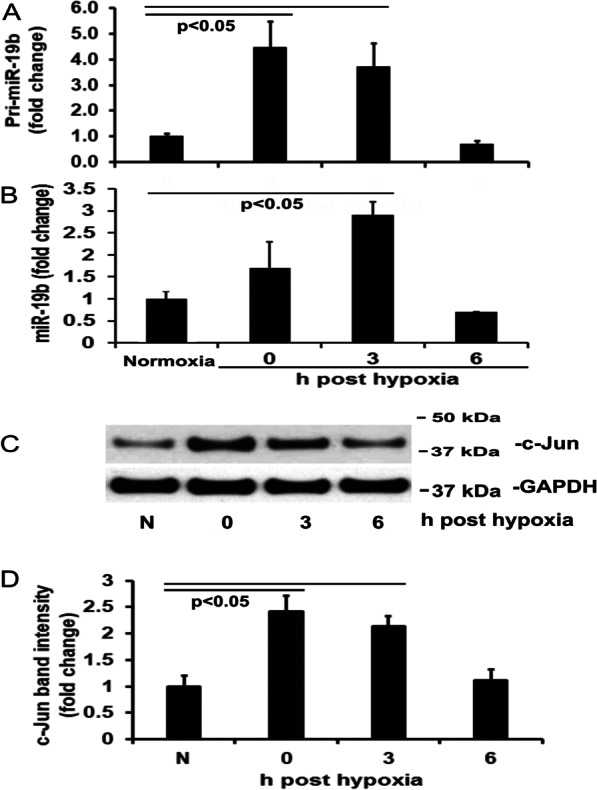
Time course of hypoxia/reoxygenation (H/R) induction on pri-miR-19b and c-Jun expression. HLMECs were exposed to normoxia only or hypoxia for 18 h then normoxia for up to 6 h. **A** Summaries of quantitative real-time PCR for pri-miR-19b. **B** Summaries of quantitative real-time PCR for mature miR-19b. **C** Representative Western blot analysis of c-Jun and GAPDH proteins. **D** Summaries of c-Jun band intensities (expressed in relative units)

We proposed that miR-19b is a miRNA responsive to H/R and thus measured pri-miR-19b and mature miR-19b. As shown in Fig. 1A and B, pri-miR-19b was significantly increased in the cells immediately after hypoxia, remained significantly high at 3 h post H/R, and returned to baseline levels at 6 h post H/R. Mature miR-19b began to increase immediately after hypoxia, it was significantly increased at 3 h post H/R but decreased to baseline levels at 6 h. The time delay may reflect the processing time from pri-miR-19b to mature miR-19b.

Cells respond to H/R by activating a number of transcription factors, including activator protein 1 (AP-1), and c-Jun protein is a component of H/R-induced AP-1 complex (Yang et al. 2013; Relja et al. 2009). Our results indicate that hypoxia alone significantly increased c-Jun protein expression and it remained elevated at 3 h post H/R compared with normoxic control (Fig. 1C and D). c-Jun expression then decreased over time during normoxia, returning to baseline levels by 6 h.

### miR-19b expression was mediated by c-Jun

**Fig. 2:**
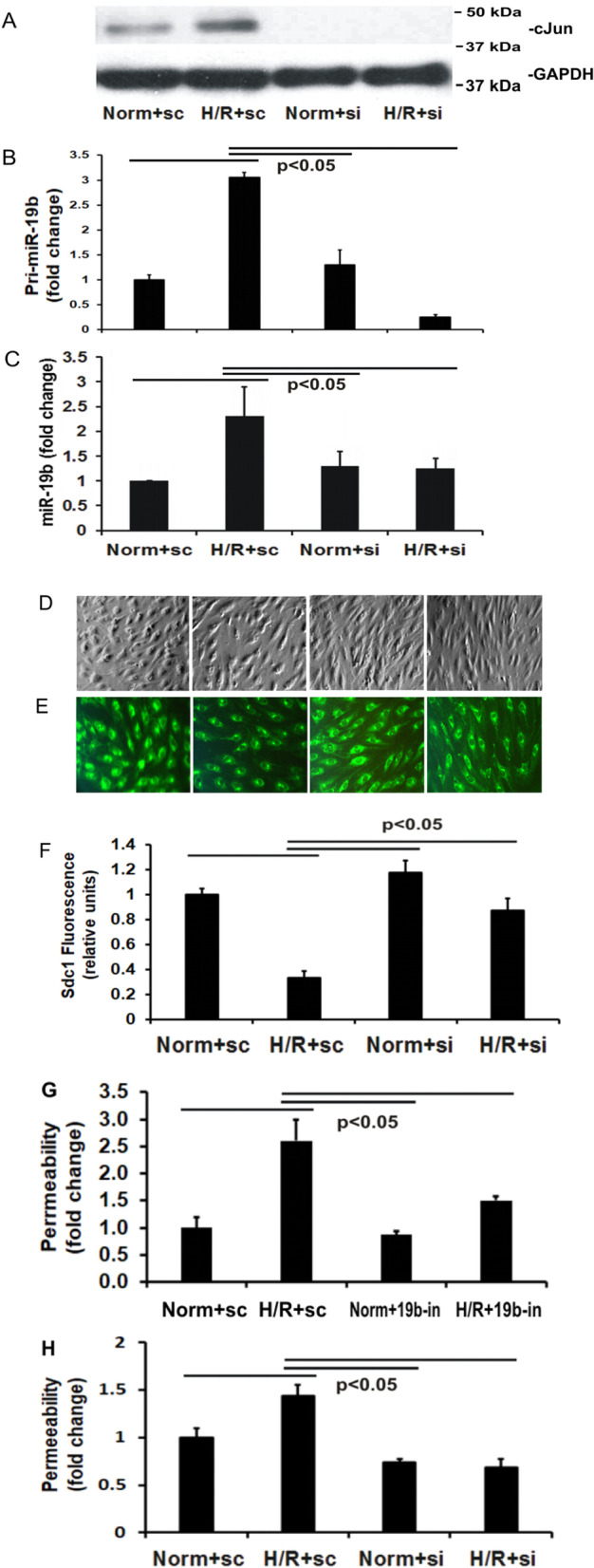
c-Jun silencing attenuates H/R-induced increases in pri-miR-19b, mature miR-19b, and cell surface syndecan-1. **A** c-Jun expression in cells transfected with c-Jun siRNA (si) or scrambled RNA (sc) then exposed to normoxia (Norm) or H/R. **B** The levels of pri-miR-19b were measured by RT-qPCR in the cells. **C** The levels of miR-19b were measured by RT-qPCR in the cells. Mean ± SE for four experiments.** D** Representative phase contrast images. **E** Cells were immunostained with anti-syndecan-1 antibody and images captured. **F** The fluorescence intensity results were presented as mean ± SE for four experiments. **G** and** H** Inhibition of either miR-19b by oligo inhibitors (19b-in) or c-Jun by siRNA (si) attenuated H/R-induced monolayer hyperpermeability. Shown were the measurements of monolayer permeability detected by FITC-dextran. Mean ± SE for four experiments

To study the regulatory effects of c-Jun on miR-19b transcription, we first used small interfering RNA (siRNA) to examine the influence of c-Jun silencing on miR-19b expression in endothelial cells. As shown in Fig. 2A, c-Jun protein was increased by H/R but substantially diminished after siRNA transfection in both normoxic and H/R cells, whereas GAPDH protein remained unchanged. c-Jun knockdown significantly reduced levels of both pri-miR-19b and mature miR-19b in normoxic and H/R cells whereas scrambled RNA cells had the expected increase in pri-miR-19b and mature miR-19b after H/R (Fig. 2B and C). These results indicate that miR-19 expression is dependent upon c-Jun.

Previously we have identified that miR-19b targeted syndecan-1 mRNA and decreased syndecan-1 expression, leading to lung vascular leakage in mice after hemorrhage shock (Wu et al. 2020). To investigate the biologic consequences of c-Jun-mediated enhancement of miR-19b expression, we first measured the expression of cell surface syndecan-1 after exposure to H/R. The representative phase contrast images are shown in Fig. 2D. Our results indicated that H/R significantly decreased cell surface syndecan-1 and this decrease was attenuated by c-Jun silencing with siRNA, confirming that c-Jun mediated syndecan-1 inhibition in H/R (Fig. 2E and F).

We further examined c-Jun/miR-19b pair involvement in regulating endothelial cell permeability. Transfection with miR-19b oligo inhibitor or siRNA targeting c-Jun attenuated H/R-induced monolayer hyperpermeability (Fig. 2G and H), supporting that c-Jun-dependent miR-19b expression mediates endothelial barrier dysfunction.

**Fig. 3:**
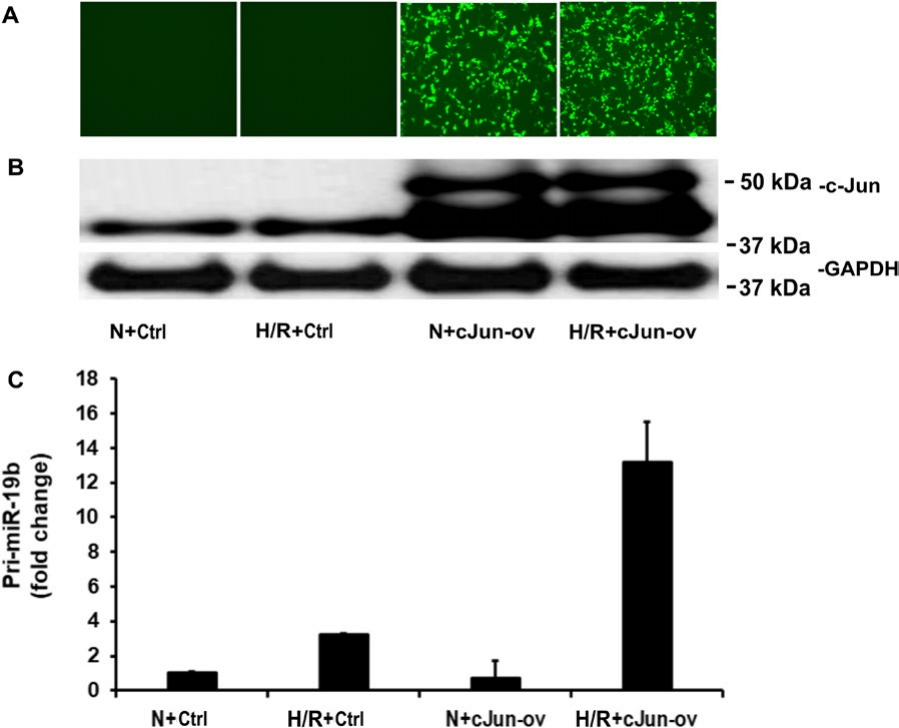
Overexpression of c-Jun enhances hypoxia-induced pri-miR-19b expression. HEK293T cells were transfected with pMIEG3-c-Jun expression vector (cJun-ov) or pMIEG3 empty vector control (Ctrl) then exposed to H/R or normoxia (N). The expression of c-Jun-GFP was examined under inverted fluorescent microscopy and images taken (**A**) and was analyzed by Western blotting (**B**). Quantitative real-time PCR for pri-miR-19b was also shown (**C**)

We next examined the effect of over-expressing c-Jun gene on the expression level of miR-19b by transfecting HEK293T cells with pMIEG3-c-Jun overexpression vector (cJun-ov) or empty vector control (Ctrl) then exposed the cells to H/R or normoxia. As shown in Fig. 3A and B, transfection of pMIEG3-c-Jun overexpression vector induced large amounts of c-Jun protein in cells as demonstrated by both immunofluorescent microscopy and Western blot analysis. We next measured the pri-miR-19b in cells after overexpression of c-Jun in normoxic and H/R conditions and found that H/R induced a threefold increase in the cells transfected with empty vector. However, H/R induced a 14-fold increase in the cells transfected with pMIEG3-c-Jun overexpression vector (Fig. 3C). These results further documented that miR-19b expression is dependent upon c-Jun (Additional file 1).

### c-Jun (AP1) is a transcript factor of the miR-19b promoter

**Fig. 4:**
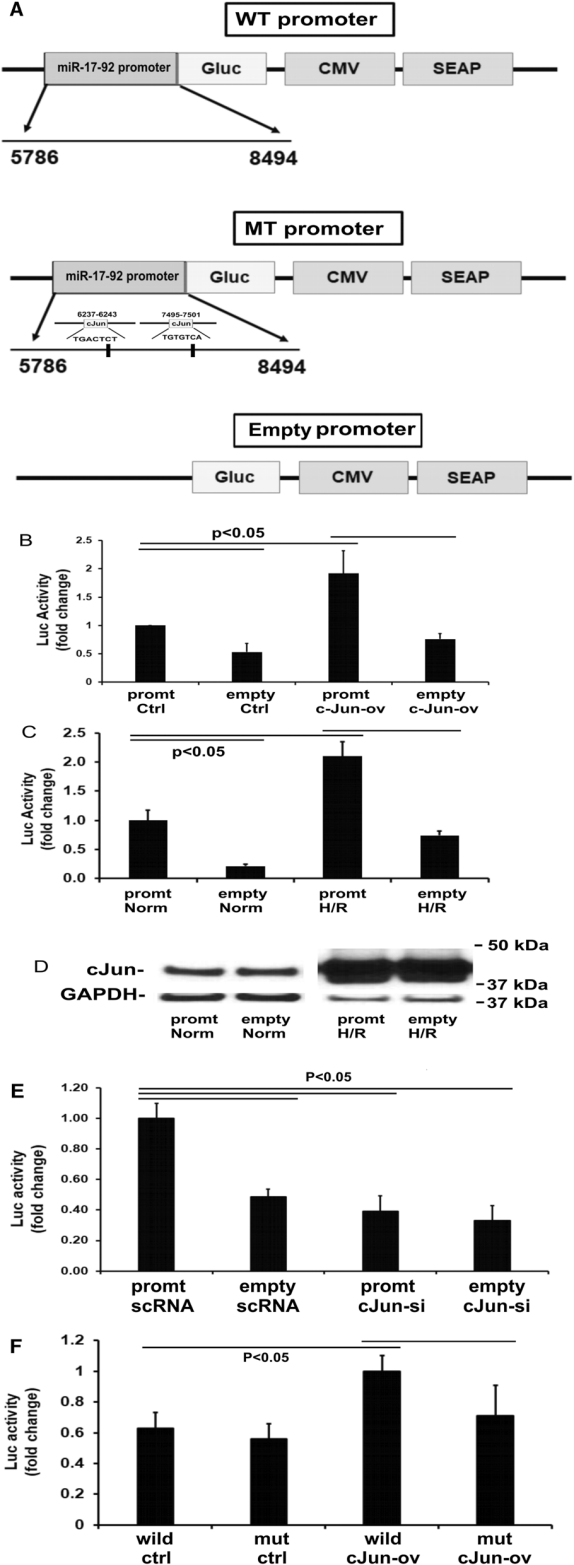
c-Jun enhances miR-17-92 promoter transcription activity. **A** miR-17-92 promoter region (positions 5786–8494 in accession# NG-032702) was inserted into pEZX-LvPG04 dual-reporter vector (WT promoter). This vector uses GLuc (Gaussia Luciferase) as the promoter reporter and SEAP (secreted alkaline phosphatase) as the internal control for signal normalization (top panel). The potential two c-Jun binding sites in the promoter were deleted (MT promoter) (middle panel). Negative control uses empty vector (Empty promoter) (bottom panel). **B** Relative luciferase (Luc) activity in the culture medium from HEK293T cells transfected with the miR-19b promoter vector (promt) or empty negative vector (empty) together with transfection with c-Jun-expression vector (c-Jun-ov) or control vector (ctrl). Mean ± SE for three experiments. **C** Relative luciferase activity in the culture medium from HEK293T cells transfected with the miR-19b promoter vector (promt) or empty negative vector (empty) then exposed to H/R or normoxia (Norm). Mean ± SE for four experiments. **D** Western blot analysis of c-Jun and GAPDH proteins. **E** Relative luciferase (Luc) activity in the culture medium from HEK293T cells transfected with the miR-19b promoter vector (promt) or empty negative vector (empty) together with transfection with c-Jun-siRNA (cJun-si) or scrambled RNA (scRNA). Mean ± SE for three experiments. **F** Relative luciferase (Luc) activity in the culture medium from HEK293T cells transfected with wild-type miR-19b promoter vector (wild) or mutated vector (mut) together with transfection with c-Jun-expression vector (cJun-ov) or control vector (ctrl). Mean ± SE for three experiments

In the following experiments, we used a luciferase reporter assay to determine if miR-19b promoter is activated by c-Jun. As shown in Fig. 4A, we prepared three types of miR-19b promoter vectors containing luciferase reporter: wild-type miR-19b promoter, mutated miR-19b promoter, and empty vectors. Compared with the cells transfected with empty vector, cells transfected with wild-type miR-19b promoter vector had significantly higher baseline luciferase activity (Fig. 4B). However, following transfection with pMIEG3-c-Jun overexpression vector, cells transfected with miR-19b promoter vector expressed twofold higher levels of luciferase activity compared with the cells transfected with empty vector (Fig. 4B). When cells were transfected with promoter vector and exposed to H/R, there was a significant increase in luciferase activity compared to empty vector H/R controls (Fig. 4C). Western blot analysis confirmed that cells exposed to H/R had increased c-Jun protein levels (Fig. 4D).

To examine if the constitutive c-Jun expression in HEK293T cells may contribute to the increase in baseline luciferase activity in cells after transfecting with miR-19b promoter vector, we thus silenced c-Jun protein expression in HEK293T cells using siRNA knockdown. As shown in Fig. 4E, HEK293 cells transfected with miR-19b promoter vector expressed about a twofold higher baseline luciferase activity compared with the cells transfected with empty vector. c-Jun knockdown reduced luciferase activity in either promoter vector-transfected cells or empty vector-transfected cells, supporting the notion that miR-19b promoter is activated by c-Jun.

We proposed that c-Jun targets miR-19b promoter DNA to initiate transcription and predicated two possible binding sites for c-Jun (AP1) binding at positions 6237–6243 (TGACTCT) and 7495–7501 (TGTGTCA). We thus produced a mutated miR-19b promoter vector by deleting these two c-Jun (AP1) binding sites. As shown in Fig. 4F, HEK293T cells co-transfected with wild-type miR-19b promoter vector plus pMIEG3-c-Jun overexpression vector demonstrated significantly increased luciferase activity compared with the cells transfected with wild-type miR-19b promoter vector plus c-Jun empty vector. However, when transfected with the mutated miR-19b promoter vector plus pMIEG3-c-Jun overexpression vector, the increase in luciferase activity was attenuated. These results further support that the miR-19b promoter is activated by c-Jun.

### c-Jun binds to the miR-19b promoter

**Fig. 5:**
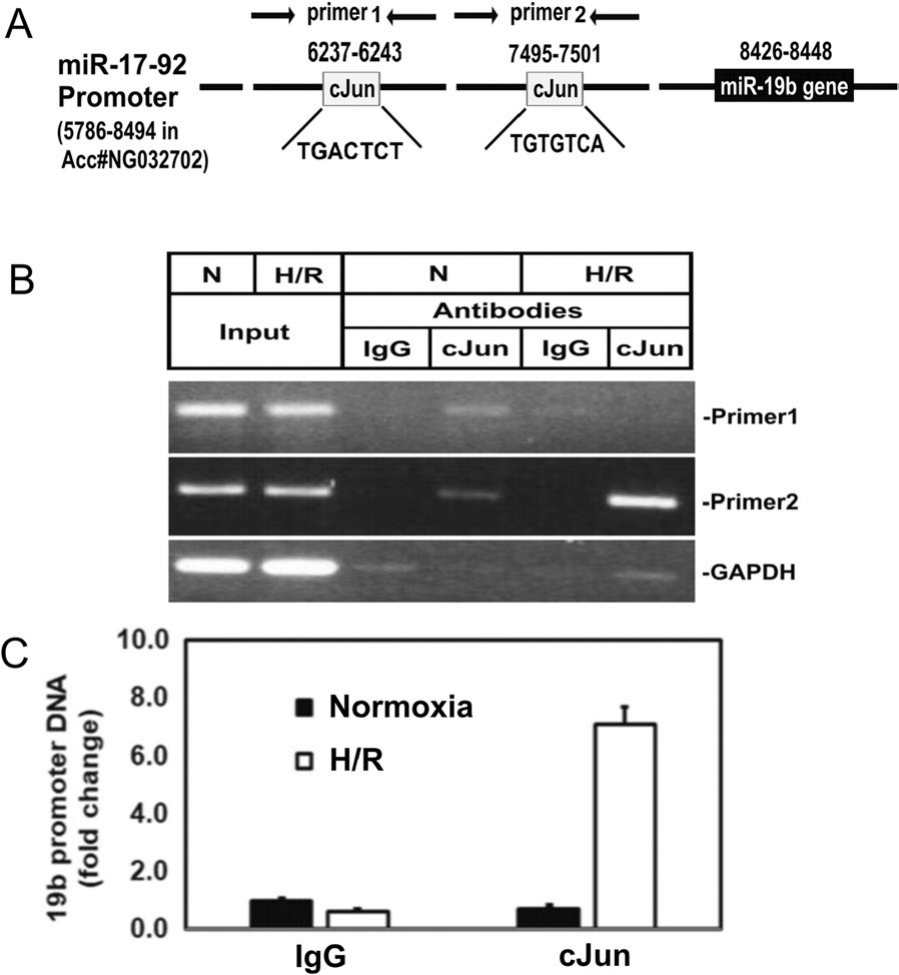
c-Jun binds to miR-17-92 promoter DNA. **A** PROMO online software was applied to predict transcription factor binding sites in the promoter region of the miR-19b gene. c-Jun was identified as a putative transcription factor with two potential binding sites in the miR-17-92 promoter at positions 6237–6243 and 7495–7502. **B** The binding activity of c-Jun to the promoter of miR-19b was determined by ChiP assay using anti-c-Jun antibody. In the cells exposed to H/R, DNA fragment around positions 7495–7501 was detected by PCR but DNA fragment around 6237–6243 was not, suggesting c-Jun mainly binds to the former binding site. No DNA fragment was detected in the chromatin immunoprecipitants isolated by IgG isotype control. **C** Summary of the DNA band intensities

Using chromatin immunoprecipitation (ChiP) assay and anti-c-Jun antibody, we next isolated the c-Jun-bound DNA and performed quantitative PCR using two pairs of primers shown in Fig. 5A. We found that c-Jun bound to position #7495–7501 but not to position # 6237–6243 and that c-Jun antibody trapped more miR-19b promoter DNA in cells exposed to H/R than in the cells exposed to normoxia (Fig. 5B), with approximately a sevenfold increase after H/R (Fig. 5C). These data therefore confirmed that c-Jun directly interacts with the promoter of miR-19b, suggesting direct regulation of miR-19b promoter activity.

### c-Jun was increased in patients with hemorrhagic shock

**Fig. 6:**
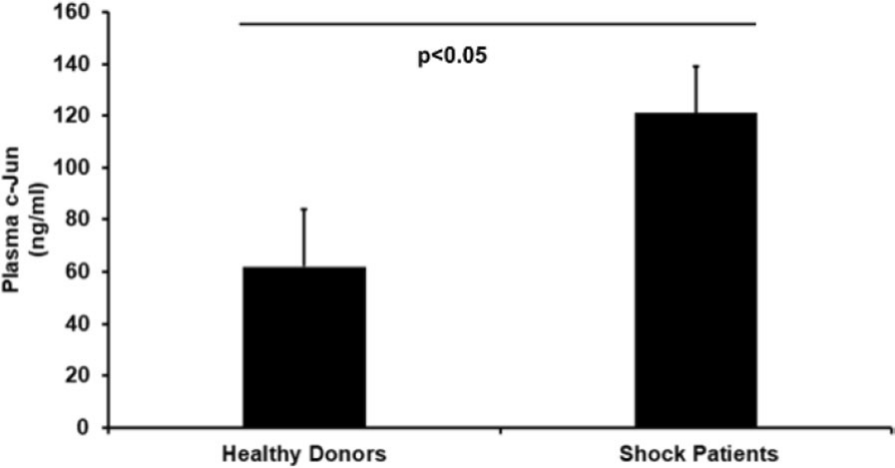
c-Jun was increased in patients with hemorrhagic shock. Plasma c-Jun was measured in 25 trauma/hemorrhage shock patients and 13 heathy donors

We measured plasma c-Jun in 25 trauma/hemorrhage shock patients and 13 heathy donors. The results indicated that hemorrhage shock patients had a significantly higher level of plasma c-Jun compared to healthy donor controls (121 ± 22 vs 64 ± 18 ng/ml, p < 0.05) (Fig. 6), indicating that c-Jun is an early responsive gene after hemorrhage shock. Demographic data is shown in Additional file 1.

## Discussion

Among multiple mechanisms involved in the pathogenesis of endothelial cell dysfunction following hemorrhagic shock, miR-19b has emerged as a novel microRNA contributing to endothelial dysfunction (Wu et al. 2020). To study the molecular mechanisms governing the response in endothelial cells after hemorrhage, we developed an in-vitro model of hemorrhagic shock which was used in the current study (Wu et al. 2020; Peng et al. 2013). We have now uncovered that miR-19b is an early responsive miRNA which is mediated by transcription factor c-Jun. Further, we found that circulating c-Jun protein was indeed increased in the plasma of severely injured patients with hemorrhage shock.

In other ischemia conditions such as stroke and myocardial infarction, miR-19b has been identified as an early responsive miRNA. For examples, miR-19b is up-regulated in response to oxygen glucose deprivation in an in-vitro model of ischemia in neuroblastoma cells (Dhiraj et al. 2013) and has been reported to be upregulated in a rodent model of cerebral artery occlusion (Jeyaseelan et al. 2008). Circulating miR-19b levels are increased in patients with acute myocardial infarction (Wang et al. 2016) and are associated with the development of diabetic cardiomyopathy (Copier et al. 2017). miR-19b has also been reported to be a downstream effector of vascular endothelial growth factor (VEGF) (Chamorro-Jorganes et al. 2016), which mediates angiogenesis and vascular leakage (Chamorro-Jorganes et al. 2016; Zhang et al. 2000). Barrier dysfunction with its associated vascular leakage is an important sequalae after hemorrhage and one we have shown is mediated by miR-19b.

Hemorrhagic shock is known to induce a global stress response following cellular hypoperfusion and hypoxia with release of pro-inflammatory cytokines into the systemic circulation. c-Jun-N-terminal kinase (JNK) is activated by cytokines and prior studies have demonstrated the important role of tissue hypoxia in its activation. We determined that circulating c-Jun protein was increased in hemorrhage shock in the present study. Hepatic JNK activation has been demonstrated in in-vivo models of liver ischemia/reperfusion (Bradham et al. 1997) and in in-vitro models of hepatocyte H/R (Crenesse et al. 2001). Additionally, McCloskey et al (2004) identified tissue hypoxia as a key factor in activating early signaling events in the liver following hemorrhagic shock as measured by JNK phosphorylation while Relja et al (2009) demonstrated liver protection by JNK inhibition following hemorrhage. There is less known, however, on the role of JNK in endothelial cells. Zakkar et al (2008) found that JNK was activated constitutively in endothelial cells at atherosusceptible sites but expressed in its an inactive form at atheroprotected sites, suggesting that a pro-inflammatory or pro-hypoxia environment is important. This is consistent with the current study in that c-Jun was present constitutively in endothelial cells, but its expression was markedly increased by hypoxia.

Upon activation, JNK regulates the activity of several transcription factors, including c-Jun, ATF-2, Elk-1, p53, and c-Myc, and is involved in the regulation of many cellular functions from proliferation to cell death. c-Jun is a major component of the AP-1 transcriptional complex. It can form either homodimers or heterodimers with other AP-1 components from the Jun family (Jun B and Jun D) or the Fos family (c-Fos, Fra-1, Fra-2, and Fos B), which directly bind to AP-1 consensus site (Angel and Karin 1991). In the present study, we documented that miR-19b expression is mediated by c-Jun which has been shown to have pathologic effects in vascular endothelial cells (Wang et al. 1999).

Indeed, our results indicate that H/R induces pri-miR-19b, mature miR-19b, and c-Jun expression over time in a comparable timeframe in human endothelial cells. c-Jun silencing by siRNA transfection reduced both pri-miR-19b and mature miR-19b expression. Conversely, c-Jun overexpression enhanced H/R-induced pri-miR-19b. Using a luciferase reporter assay, we observed that in cells transfected with vectors containing the wild-type miR-19b promoter and luciferase reporter, c-Jun overexpression or H/R significantly increased luciferase activity. c-Jun knockdown reduced the luciferase activity in these cells, suggesting that the miR-19b promoter is directly activated by c-Jun. Finally, as assessed by chromatin immunoprecipitation assay, we confirmed that c-Jun binds to the promoter DNA of miR-19b and H/R significantly increased this interaction.

Our previous study demonstrated that miR-19b targeted syndecan-1 mRNA and decreased syndecan-1 expression, leading to lung vascular leakage in mice after hemorrhage shock. Overexpression of miR-19b reduced syndeccan-1 expression and increased permeability in pulmonary endothelial cells after H/R (Wu et al. 2020; Chipman et al. 2021). The present study further confirmed that c-Jun mediates miR-19b increase, cell surface syndecan-1 decrease, and endothelial barrier dysfunction in H/R. Additionally, circulating c-Jun protein was increased in the plasma of severely injured patients with hemorrhage shock.

## Conclusion

Transcription factor c-Jun is inducible by hypoxia/reoxygenation, and it binds to and activates the miR-19b promoter. Using an in-vitro model of hemorrhagic shock, our findings identified a novel cellular mechanism whereby hypoxia/reoxygenation increases miR-19b transcription by inducing c-Jun and leads to syndecan-1 decrease and endothelial cell barrier dysfunction. Our results also show that c-Jun is increased in patients with hemorrhagic shock. Thus, our study supports that miR-19b could be a potential therapeutic target for hemorrhage shock.

## Supplementary Information

**Additional file 1. Additional Table 1:** Trauma/hemorrhage shock patient demographics

## Data Availability

The datasets used analyzed during the current study are available from the corresponding author on reasonable request. Further de-identified patient data can be obtained via inquiry to the corresponding author.

## Abbreviations

miR-19b: MicroRNA-19b
H/R: Hypoxia/reoxygenation
JNK: C-Jun N-terminal kinase
AP-1: Activating protein-1
HUMECs: Human lung microvascular endothelial cells
EBM-2: Endothelial basic medium-2
Gluc: Gaussia luciferase
SEAP: Secreted alkaline phosphatase
FITC: Fluorescein isothiocyanate
ChIP: Chromatin immunoprecipitation
siRNA: Small interfering RNA
