# Supplementary material for: c-Jun-mediated miR-19b expression induces endothelial barrier dysfunction in an in vitro model of hemorrhagic shock
Source: Mol Med. 2022 Oct 12;28:123. doi: 10.1186/s10020-022-00550-0 (PMC9558999; doi:10.1186/s10020-022-00550-0)
Supplement: Supplementary file 1 — Additional file 1. Additional Table 1: Trauma/hemorrhage shock patient demographics [file 10020_2022_550_MOESM1_ESM.docx]

**Additional Table 1: Trauma/hemorrhage shock patient demographics**

| **Parameter (n= 25)** | **Value** |
| --- | --- |
| Age (years) | 45 ±18 |
| Male sex, n (%) | 20 (80%) |
| Mechanism |  |
| Blunt | 17 (68%) |
| Penetrating | 8 (32%) |
| Injury Severity Score | 26 (17-34) |
| Systolic blood pressure on arrival | 66 (60-75) |
| Total blood in 24hours (units) | 18 (9-34) |
| Exploratory laparotomy | 8 (32%) |
| Thoracotomy | 3 (12%) |
| Ventilator days | 4 (2-10) |
| ICU days | 5 (2-12) |
| Hospital length of stay | 14 (7-26) |
| Mortality, n (%) | 2 (8%) |
